# ELDERLY MOUSE DETRUSOR MAINTAINS ITS PEAK FORCE OF CONTRACTION OVER TIME

**DOI:** 10.1093/geroni/igac059.2451

**Published:** 2022-12-20

**Authors:** Nadav Mortman, Ramalakshmi Ramasamy, Phillip Smith

**Affiliations:** University of Connecticut School of Medicine, FARMINGTON, Connecticut, United States; UConn Health, Farmington, Connecticut, United States; UConn Health, Farmington, Connecticut, United States

## Abstract

Aging is a risk factor for urinary dysfunction. Detrusor weakening has been thought to contribute to the old bladder phenotype. Animal detrusor strips have been used to study bladder function. Strength of contraction in detrusor strips depends on length. This is referred to as the “length-tension relationship.” The aim of our study was to investigate this relationship in a mouse model by comparing the length-tension relationship in old and mature mice. We hypothesize that aging is associated with no change in active detrusor tension capability, however maximal tension will occur at longer lengths.We used two groups of male C57/Bl6 mice for this study, mature 11-12 month old mice and old 22-23 month old mice. Longitudinal intact bladder strips were harvested and placed in a vertical tissue bath between tension recording transducer hooks. Passive tensions and KCl induced contraction tensions for step-wise increments of stretch were observed.In the old group, normalized strip length at the point of maximum active tension was increased by ~13% on average as compared to the mature group with a statistically significant difference (P value 0.0171). Interestingly, the maximum active tension between groups did not differ by age. In conclusion, detrusor from old mice achieve similar maximum active tensions as that from mature mice, however at an increased length. This finding argues against a common belief that the bladder weakens with age. Instead, the aging bladder may adapt to increased filling volumes with an ability to operate at a similar strength of a younger bladder.

